# The catalytic mechanism, metal dependence, substrate specificity, and biodiversity of ribonuclease H

**DOI:** 10.3389/fmicb.2022.1034811

**Published:** 2022-11-21

**Authors:** Jing Pang, Qinyu Guo, Zheng Lu

**Affiliations:** Department of Biology, Guangdong Provincial Key Laboratory of Marine Biotechnology, Institute of Marine Sciences, Shantou University, Shantou, China

**Keywords:** RNase H, R-loops, metal choice, biodiversity, HIV drug, RNA/DNA hybrids

## Abstract

Ribonucleoside monophosphates are inevitably misincorporated into the DNA genome inside cells, and they need to be excised to avoid chromosome instability. Ribonucleases H (RNases H) are enzymes that specifically hydrolyze the RNA strand of RNA/DNA hybrids or the RNA moiety from DNA containing a stretch of RNA, they therefore are required for DNA integrity. Extensive studies have drawn a mostly clear picture of the mechanisms of RNase H catalysis, but some questions are still lacking definitive answers. This review summarizes three alternative models of RNase H catalysis. The two-metal model is prevalent, but a three-metal model suggests the involvement of a third cation in catalysis. Apparently, the mechanisms underlying metal-dependent hydrolyzation are more complicated than initially thought. We also discuss the metal choices of RNases H and analyze how chemically similar cations function differently. Substrate and cleavage-site specificities vary among RNases H, and this is explicated in detail. An intriguing phenomenon is that organisms have diverse RNase H combinations, which may provide important hints to how *rnh* genes were transferred during evolution. Whether RNase H is essential for cellular growth, a key question in the study of *in vivo* functions, is also discussed. This article may aid in understanding the mechanisms underlying RNase H and in developing potentially promising applications of it.

## Introduction

Ribonucleoside monophosphates (rNMPs) are always incorporated into DNA in all living cells. RNA primers are required to initiate the synthesis of both the leading strand and Okazaki fragments on the lagging strand during genome replication (Yan et al., 2018). DNA replicases lack absolutely perfect nucleotide selectivity and occasionally misincorporate ribonucleotides into DNA (Zhou et al., 2021). The rNMPs in DNA could be toxic to cells if they are not processed. Excess errant misincorporation of ribonucleotides by DNA polymerases is harmful to cells: chromosome instability increases because the reactive 2′-OH of rNMPs may leave 2′,3′ cyclic phosphate on DNA and cause damage (Oivanen et al., 1998). The shape of the DNA backbone can be altered by inserted rNMPs (Potenski and Klein, 2014). Besides, RNA primers of Okazaki fragments must be removed during lagging strand DNA synthesis, otherwise they might lead to conditional lethality in cells by hindering the progression of the replication fork (Qiu et al., 1999a). R-loops, which are typically generated as a by-product of transcription when nascent mRNA molecules hybridize with the template DNA, are another cause of DNA replication stalling and cell lethality (Gan et al., 2011; Lang et al., 2017). Therefore, it is necessary for living organisms to remove RNA moieties from DNA. A ubiquitous cellular mechanism for doing this involves ribonucleases H (RNases H).

RNases H belong to the nucleotidyl-transferase superfamily which rely on divalent cations to catalyze nucleophilic substitution reactions, they specifically hydrolyze rNMPs or stretches of RNA in a diverse range of nucleic acids, such as RNA/DNA hybrids, R-loops, and double-stranded DNA with an embedded single ribonucleotide, etc. (Nowotny et al., 2005; Yang et al., 2006). Based on amino acid sequences, structures, and substrate specificities, RNase H enzymes are separated into two types: type 1 (generally denoted RNase HI in bacteria and H1 in eukaryotes) and type 2 (RNase HII, HIII in bacteria and H2 in eukaryotes; Cerritelli and Crouch, 2009; Tadokoro and Kanaya, 2009). They are required in DNA replication and repair processes in organisms such as bacteria and yeast, and RNase H1 knockout results in embryonic lethality in mice, defects in RNase H2 lead to autoimmune disorders, Aicardi–Goutières syndrome (AGS), systemic lupus erythematosus, and skin and intestinal cancers in humans (Arudchandran et al., 2000; Cerritelli et al., 2003; Crow et al., 2006; Tadokoro and Kanaya, 2009). Besides, human immunodeficiency virus-1 (HIV) reverse transcriptase possesses both DNA polymerase and RNase H activity, the latter of which is necessary for such a reverse transcriptase to convert single-stranded viral RNA genome into double-stranded DNA within host cells. RNase H activity is thought to be an up-and-coming target for creating anti-AIDS drugs (Tisdale et al., 1991; Klumpp et al., 2003; Shaw-Reid et al., 2005).

RNases H generally function as sequence-nonspecific endoribonucleases and have only been reported as an exoribonuclease when processing substrates with a 3′-DNA overhang side (Lee et al., 2022). Compared to known ribonucleases, RNases H are unusual for the property of divalent metal ion–dependent catalysis, which is particularly prevalent among DNases (Keck et al., 1998). In this review, we describe the three different models of catalysis, metal ion choices, substrate specificities, and biodiversity of prokaryotic and eukaryotic type 1 and type 2 RNases H. We also discuss the chemical characteristics of cations and how they affect the activity of RNases H, and we address a few areas where outstanding questions exist. This article may be helpful in further understanding the molecular mechanisms underlying metal-dependent RNase H catalysis and the evolutionary significance of diverse combinations of RNases H.

## Models of RNase H catalysis

Type 1 and type 2 RNases H hydrolyze substrates by similar mechanisms, they share a common folding motif (called RNase H-fold), and active-site residues of both types show analogous spatial configurations (Tadokoro and Kanaya, 2009). RNase H activity strictly depends on divalent-cation cofactors. It has long been a subject of debate whether RNase H needs one, two, or even more metal ions for catalysis. This is because the number of metal ions in crystal structures of different RNases H with and without substrates varies: some have one ion, whereas others have two or three (Katayanagi et al., 1993; Goedken and Marqusee, 2001; Tsunaka et al., 2005; Nowotny and Yang, 2006; Yang et al., 2006).

**Figure 1:**
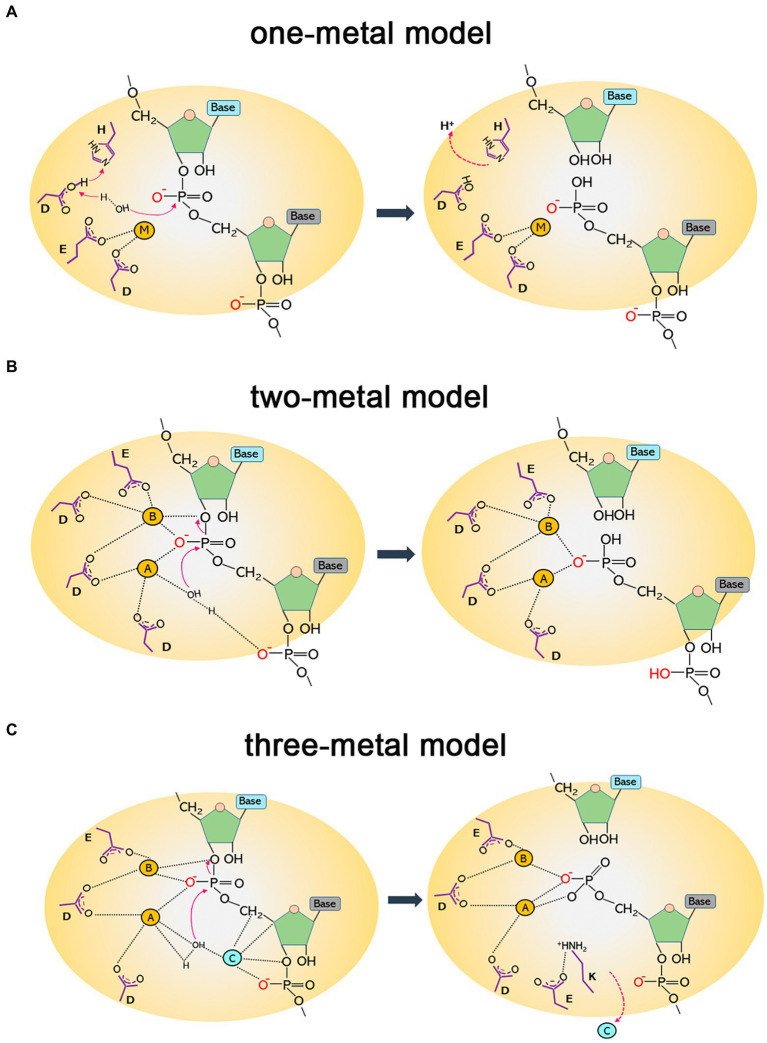
Diagrams of catalytic mechanisms of RNases H. **(A)** One-metal model. The imidazole group of a histidine residue promotes the deprotonation of the acidic side chain of an aspartate, which further deprotonates a water molecule. The nucleophilic hydroxyl produced attacks the phosphodiester bond, leading to hydrolytic cleavage. The single metal ion (M) is bound to two conserved carboxylates (aspartate and glutamate) to stabilize a transition state intermediate. **(B)** Two-metal model. The hydrolysis of the P-O3′ bond relies on two metal ions. Metal ion A, coordinated by two aspartates, activates a water molecule to attack the scissile phosphate as a nucleophile; metal ion B is shared between an aspartate and a glutamate and facilitates the phosphoryl transfer reaction. **(C)** Three-metal model. Besides the two canonical metals that function as in the two-metal model, a third ion (C) is transiently bound to synergize with metal A and activate water for a nucleophilic attack. It departs immediately after cleavage. A conserved lysine may assist in aligning the substrate to capture transient cations for catalysis. It forms a salt bridge with a glutamate to facilitate the removal of cations and product release.

To date, three alternative models of the enzymatic mechanisms of RNases H have been proposed: the one-metal, two-metal, and three-metal models. The one-metal-ion model is thought to be a general base carboxylate-hydroxyl relay mechanism. For type 1 RNases H, the imidazole side chain of a histidine residue facilitates the deprotonation of a conserved aspartate, which further activates a water molecule to generate a nucleophilic hydroxyl ion attacking the scissile phosphate. The single metal ion is bound by two conserved acidic residues (aspartate and glutamate) to stabilize a transition state intermediate by generating an outer sphere-coordination complex (Kanaya et al., 1990; Oda et al., 1993; Keck et al., 1998; Figure 1A). Type 2 RNases H lack the conserved histidine that triggers the deprotonation of the water nucleophile and might use a carboxylate residue in place of the histidine (Ohtani et al., 1999b).

In the two-metal-ion catalysis model, the cations are separated by ∼4 Å. One metal activates the water that acts as the attacking hydroxyl nucleophile in hydrolysis, and the other metal stabilizes the transition state—the penta-covalent phosphorane intermediate—by serving as an electrophilic catalyst binding to the bridging and non-bridging oxygen atoms of the scissile phosphate diester. Four negatively charged carboxylate residues (DEDD for HI/1 and HII/2 and DEDE for HIII) are conserved in the active sites to fully coordinate the two divalent cations (Klumpp et al., 2003; Nowotny et al., 2005; Nowotny and Yang, 2006; Yang et al., 2006; Figure 1B). This two-metal model is more widely accepted than the one-metal model. However, from the time-resolved crystallographic structure of *Bacillus halodurans* RNase H1 complexed with an RNA/DNA hybrid substrate, a transiently bound third divalent metal ion was observed and was proved to be required for catalysis (Figure 1C). This confirms the existence of a three-metal-ion-dependent catalytic mechanism for the RNase HI/1 superfamily, and this model is supported by other studies as well (Ho et al., 2010; Gao and Yang, 2016; Samara and Yang, 2018; Yang and Gao, 2018; Chang et al., 2022).

## Metal choices of RNases H

No matter which model of catalysis is adequate, RNase H activity always depends on divalent metal ions, although the metal preference may vary widely. In general, Mg^2+^ or Mn^2+^ is normally the best choice, while Co^2+^, Zn^2+^, Ni^2+^, or even Cd^2+^ can support catalysis for some RNases H, such as those from *Archaeoglobus fulgidus*, *Pyrococcus kodakaraensis*, and *Bacillus subtilis*, but usually at a low level. Ca^2+^ can stabilize the ES complex but basically inhibits RNA hydrolysis (Nowotny and Yang, 2006; Rosta et al., 2014). We summarize metal cofactors for some RNases H in Table 1. Although Mg^2+^ and Mn^2+^ have very similar chemical properties, generally only one of the ions is selected as the optimum cofactor for an RNase H; the other may sustain activity but relatively weakly, although the reason for this remains to be clarified. RNases H from different species may have different metal ion preferences when they cleave the same kind of substrate *in vitro* (Table 1); however, a given RNase H may not favor the same metal ion as the cofactor when degrading various substrates (Table 1).

**Table 1:** Metal choices of RNases H.

RNase H	Substrate	Metal preference	Source
*Mycobacterium smegmatis* RNase HI	RNA/DNA	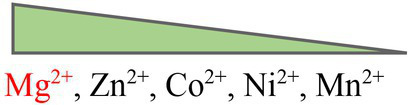	Richa Gupta et al. (2017)
*Escherichia coli* RNase HI	RNA/DNA	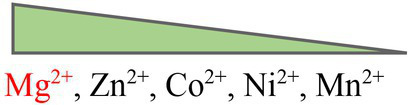	Uchiyama et al. (1994)
*Shewanella* sp. SIB1 RNase HII	RNA/DNA	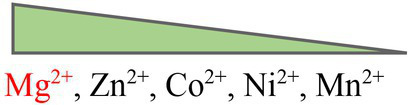	Chon et al. (2006b)
*Chlamydia pneumonia* RNase HII	RNA/DNA	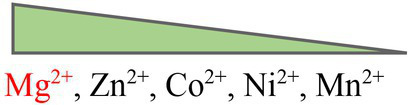	Pei et al. (2005)
*Archaeoglobus fulgidus* RNase HII	RNA/DNA	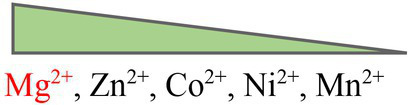	Chai et al. (2001)
*Pyrococcus kodakaraensis* KOD1 RNase HII	RNA/DNA	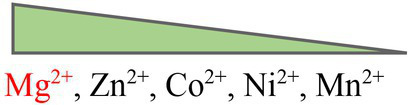	Haruki et al. (1998)
*Aeropyrum pernix* RNase HII	RNA/DNA	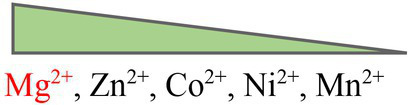	Hou et al. (2012a)
*Bacillus subtilis* RNase HII	Dr_4_D/DNA	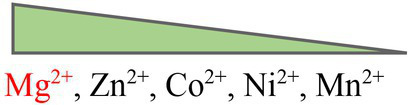	Randall et al. (2018)
	Dr_1_D/DNA	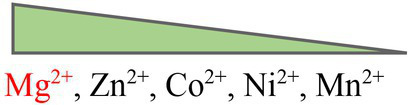	
*Bacillus subtilis* RNase HIII	RNA/DNA	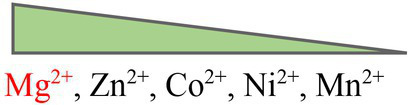	Randall et al. (2018)
	Dr_4_D/DNA	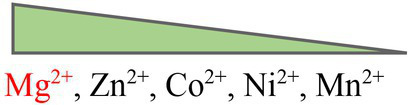	
	Dr_1_D/DNA	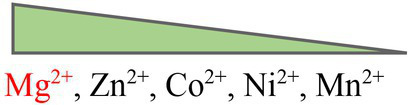	

The optimal metal cofactor is in red; edges represent different levels of activity dependent on metal ions. Dr_1_D/DNA is the substrate of dsDNA that contains a single ribonucleotide. Dr_4_D/DNA is dsDNA with four embedded ribonucleotides.

With regard to why RNases H have different metal preferences for catalysis (Mg^2+^, Mn^2+^, or Co^2+^), there is not yet any well-defined explanation. The catalytic core of all RNases H is the same. Crystal structures of Ca^2+^ (a noncatalytic cation)- and Mg^2+^-bound RNase H2-substrate complexes do not seem to differ from each other, similar to other nucleases and polymerases (Viadiu and Aggarwal, 1998; Lee et al., 2005; Rychlik et al., 2010), so it seems hard to interpret the question from the point of view of protein structure. Other factors to consider include (a) the fact that the particular physicochemical properties of a metal ion may impact its engagement in catalysis during biological processes; (b) the fact that the natural abundance of cellular Mg^2+^ over other metal ions can offer cells more opportunities to use it as a native cofactor; and (c) the fact that because organisms live in diverse habitats where the availability of metal ions for enzymatic activity differs, the enzyme may have evolved to natively adopt a metal cofactor as the organism has adapted to its surroundings (Lu et al., 2018; Lu and Imlay, 2019, 2021).

Magnesium, by virtue of its superior abundance (intracellular concentration: 15–25 mM in total, <2 mM in free form in bacteria such as *Escherichia coli*) and unique properties, is a mandatory cofactor coordinating the catalytic pocket of RNases H of most biological origins (Outten and O’halloran, 2001; Ando et al., 2021). It shows higher affinity for oxygen-donor ligands (carboxylates, phosphates, etc.) than many cations such as calcium, and the ligands are always hexa-coordinated and form a classical octahedral conformation which is more rigid than those of other ions (Wolf and Cittadini, 2003; Babu et al., 2013). It has a relatively small atomic radius, the hydration radius is 400 times greater than the dehydration radius, and it has high hydration energy and the highest charge density of all biological cations (Moomaw and Maguire, 2008; Babu et al., 2013).

Manganese is another candidate cofactor of RNases H. Its ionic properties, such as the atomic radius, coordination distance, and ligand number, are similar to those of Mg^2+^. However, compared to the highly stringent Mg^2+^, Mn^2+^ is more tolerant of both mutations in enzymes and distortions in substrates and the requirements of the coordination geometry of Mn^2+^ are relatively flexible, which allows certain enzymes to exploit it as the cofactor to cut otherwise unfavorable substrates with reduced specificity (Kunkel and Loeb, 1979; Cirino et al., 1995; Yang et al., 2006). For example, RNase HII from *Thermotoga maritima* specifically digests Okazaki fragment–like substrates using Mg^2+^ as the cofactor, but it does not show such a substrate preference when cofactored by Mn^2+^ (Rychlik et al., 2010). *Pyrococcus furiosus* RNase HII shows Mn^2+^-supporting activity on RNA–RNA duplexes, which are atypical substrates for RNase H (Kitamura et al., 2010).

Calcium is a potent competitor of magnesium in biological systems. However, its hydration radius is just 25 times larger than its dehydration radius, and this ratio is much smaller than that for magnesium (Moomaw and Maguire, 2008). Moreover, hydrated Ca^2+^ has a high ligand exchange constant, so it transfers more easily between two ligands. Its coordination number is 6–10, with more flexible octahedra than Mg^2+^ (Maguire and Cowan, 2002). Another reason why Ca^2+^ rarely supports the activity of enzymes such as RNase H is that Ca^2+^ easily precipitates negatively charged biological macromolecules such as DNA, RNA, and some acidic proteins. Therefore, it is hard for organisms to keep Ca^2+^ and macromolecules together in solution without sedimentation, which leads to restricted selectivity in utilizing Ca^2+^ (Frausto Da Silva and Williams, 2001).

The highly conserved DED residues in the catalytic pocket of RNases H act as general bases liganding metal ions. Mg^2+^ binds the first two carboxylates (Figure 1B), while Ca^2+^ is further liganded by two oxygen atoms of the carboxylate group of the third Asp. This tightens the binding of calcium to the enzyme but also prevents the aspartate from acting as a common base to be a proton/hydrogen bond acceptor (Moomaw and Maguire, 2008). The net charge of the metal complex is −1 instead of 0, which leads to the cancellation of enzymatic activity (Moomaw and Maguire, 2008). Rosta et al. (2014) studied the mechanism underlying Ca^2+^ inhibition in *Bacillus halodurans* RNase H1 activity using molecular simulations. They found that Ca^2+^ substitution of active-site Mg^2+^ brought about increased change in the reaction barrier and ion coordination geometry; it was inferred that Ca^2+^ inhibition of RNase H activity might be due to the fact that this ion shows a differential ability for metal ligand charge transfer compared to Mg^2+^. Nevertheless, the inhibitory effect of Ca^2+^ on RNase catalysis does not always hold. *E. coli* RNase I (a single-stranded RNA endonuclease) has strong calcium-dependent activity on double-stranded RNA (dsRNA), and the cofactor cannot be substituted by other ions (Grunberg et al., 2021).

Finally, from a stereochemical point of view, nickel is more similar to Mg^2+^, having the same size, water exchange rate, and geometric configuration, and it is a good candidate for magnesium transport systems. However, this ion tends to bind nitrogen donors rather than oxygen donors, and thus it is disqualified from competing with Mg^2+^ inside cells (Wolf and Cittadini, 2003).

## Substrate specificities of RNases H

There are three types of RNase H substrates: RNA/DNA hybrids or RNA/DNA hybrid-containing R-loops, RNA–DNA/DNA (Okazaki-like substrates), and DNA-rN_(n)_-DNA/DNA chimeric substrates. Type 1 RNases H can cleave RNA/DNA hybrids and RNA–DNA/DNA substrates but require DNA–RNA–DNA/DNA chimeric substrates to contain at least four ribonucleotides (Cerritelli and Crouch, 2009; Tadokoro and Kanaya, 2009). The crystal structures of type 1 RNases H complexed with RNA/DNA hybrid substrates show that the DNA strand of the substrate must be twisted to be recognized by the phosphate-binding pocket of RNase H1/HI (Nowotny et al., 2005); therefore, the DNA strand needs to have a certain flexibility. In one study, when one deoxyribonucleotide of the DNA strand in RNA/DNA hybrids was chemically modified (i.e., the flexibility of free twisting of the DNA chain was reduced), the ribonucleotides paired to the modified DNA were no longer hydrolyzed by human RNase H1 (Lima et al., 2007). Therefore, RNases H must not only recognize the strand of RNA but also correctly identify the DNA strand in the RNA/DNA hybrid, otherwise activity will be compromised.

Although type 2 RNases H also cleave RNA/DNA hybrids and RNA–DNA/DNA substrates, their cleavage-site specificities obviously vary from those of the type 1 enzymes (Cerritelli and Crouch, 2009). RNases H2/HII cut the dsDNA containing four chimeric ribonucleotides at the position of the two ribonucleotides in the middle, thus leaving one ribose at both ends. However, RNases H1/HI function at the two ribonucleotides near the 5′-terminus of the DNA strand. When hydrolyzing RNA/DNA heterozygous substrates, type 1 RNases H also cut ribonucleotides close to the 5′-end of the RNA chain, but the cleavage site of H2/HII is near the 3′-end (Cerritelli and Crouch, 2009).

Type 1 and type 2 RNases H hydrolyze substrates in different ways *in vitro*, which suggests that the two types of RNases H may act on different substrates *in vivo*. Bubeck et al. observed under fluorescence microscopy that human RNase H2 but not H1 localizes to the replication foci in the nucleus, which indicates that only RNase H2 is involved in genome replication/repair in mammalian cells (Bubeck et al., 2011). Instead, eukaryotic type 1 RNases H is necessary for mitochondrial DNA replication (Busen et al., 1977; Turchi et al., 1994; Cerritelli et al., 2003). With regard to why RNases H1 and H2 have overlapping functions to degrade RNA/DNA hybrids or R-loops inside cells, Zimmer and Koshland, (2016) confirmed that the hybrid removal activities of both enzymes have distinct functions in yeast. RNase H2 but not H1 is the major enzyme that hydrolyzes R-loops and protects cells from large-scale chromosome instability.

Eder and Walder, (1991) were the first to show RNase H’s incision on dsDNA with a single embedded ribonucleotide when they reported that human RNase H2 can cleave DNA-rN_1_-DNA/DNA substrates. Subsequent reports proved that RNases H2/HII from various prokaryotic and eukaryotic sources can also incise such substrates (Frank et al., 1998; Haruki et al., 1998; Ohtani et al., 1999a; Haruki et al., 2002; Rydberg and Game, 2002). Some bacterial RNases HIII also cut these substrates, most efficiently with Mn^2+^ as the cofactor (Lu et al., 2012b; Randall et al., 2018). All type 1 RNases H lack this cleavage ability, and therefore a significant difference in substrate specificities exists between type 1 and type 2 RNases H.

It is noteworthy that, according to Cerritelli and Crouch, (2009), most *in vitro* studies of the cleavage activity of RNases H have used substrates labeled with fluorescent groups at the 5′-end or 3′-end of the nucleic acid chain. When the substrate is adequately cleaved, the bands of one or more cleavage products can be observed; however, when the cleavage site is close to the end of the fluorescent label, the cleavage of other sites might be blurred. The result is that RNase H appears to prefer the cleavage site, when in actuality this may not be the case; the enzyme probably incises at other sites.

## Two prokaryotic type 2 RNases H: RNases HII and HIII

**Figure 2:**
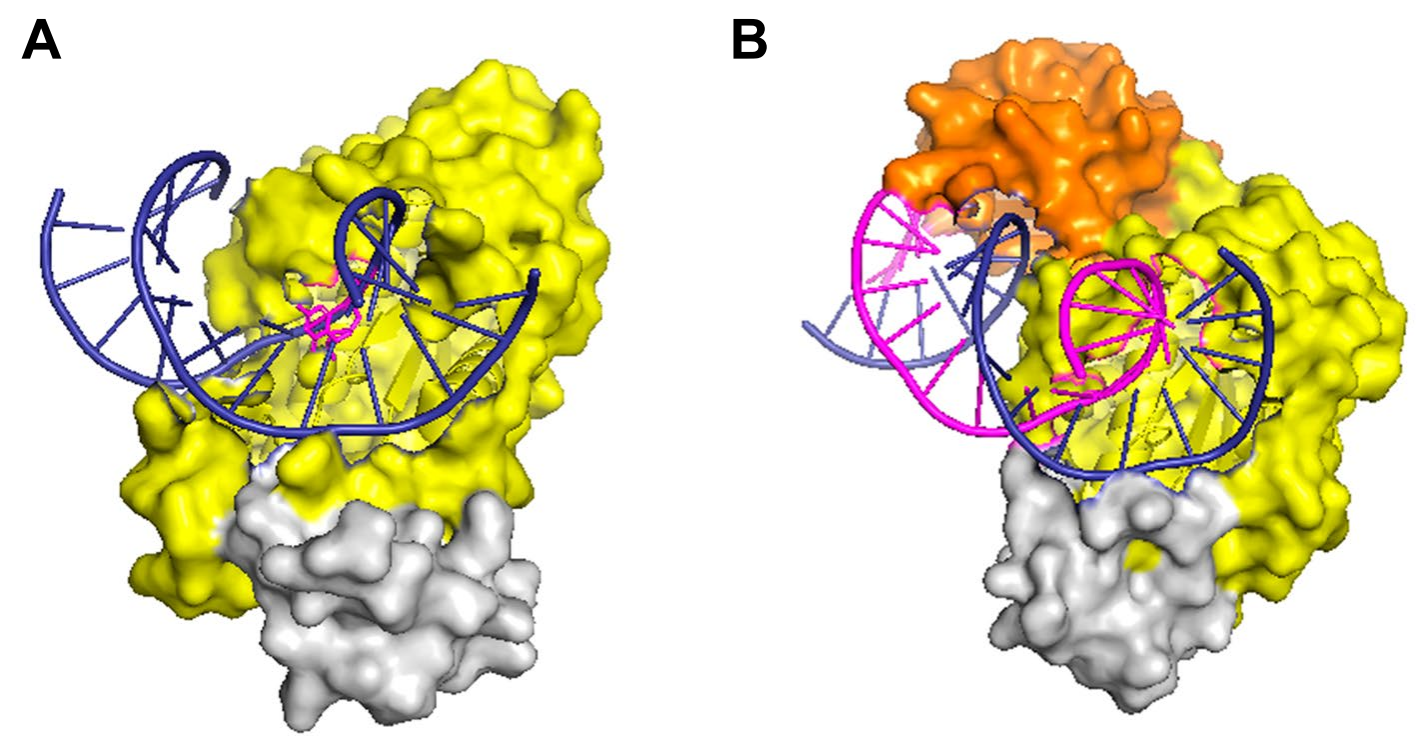
Structures of RNase HII and HIII in complex with a nucleic acid substrate in surface representation. **(A)** Structure of *T. maritima* RNase HII complexed with a dsDNA containing a single ribonucleotide [protein data bank (PDB) code 3O3F]. **(B)** Structure of the complex between *Thermovibrio ammonificans* RNase HIII and an RNA/DNA hybrid (PDB code 4PY5). In both panels, the “RNase H-fold” structure is shown in yellow, the C-terminal helix domain is colored gray, and the N-domain of RNase HIII is shown in orange. Nucleic acid substrates are shown in cartoon representation with DNA strands in deep blue and RNA in purple. The figure was prepared using Pymol.

RNases HII and HIII of prokaryotes are classified as type 2 RNases H based on their amino acid homology. RNase HII exists widely in the microbial world, whereas HIII is only present in a few bacteria. By studying the structure of *Geobacillus stearothermophilus* RNase HIII, Chon et al. (2006a) proved that HIII is not only homologous to HII in primary structure but also closely related stereo-structurally. Both adopt the RNase H-fold as the main structure of the catalytic domain. However, unlike HII, HIII has an independent subdomain (about 80 amino acids) at the N-terminus; such a structure is absent in RNases HII (Figure 2). The N-terminal domain participates in substrate binding (Chon et al., 2006a), but in the docking model of RNase HIII complexed with an RNA/DNA heterozygous substrate, this domain is not in actual contact with the substrate, and the distance between them is great. Miyashita et al. (2011) believed that the N-terminal domain of RNase HIII may bend down to approach and bind to the substrate. Figiel and Nowotny resolved the crystal structure of RNase HIII in complex with an RNA*/*DNA substrate and proved that the N-terminus of the enzyme is involved in substrate binding (Figiel and Nowotny, 2014; Figure 2).

Despite the homologous catalytic domains of RNases HII and HIII, the enzymatic properties and substrate specificity of HIII are similar to HI but different from HII (Ohtani et al., 1999a; Haruki et al., 2002; Chon et al., 2004, 2006a; Liang et al., 2007). For example, RNases HII can incise dsDNA substrates embedded with a single ribonucleotide, but neither HI nor HIII (with a few exceptions) can cut these substrates (Cerritelli and Crouch, 2009; Tadokoro and Kanaya, 2009). Rychlik et al. (2010) analyzed the structure of the complex formed by *T. maritima* RNase HII and DNA-rN_1_-DNA/DNA substrates and proved that the G_21_R_22_G_23_ motif and Y_163_ residue are responsible for recognizing the monoribonucleotide in dsDNA. The GR (K) G motif for substrate recognition is highly conserved in HII and HIII. However, the tyrosine that can interact with the single ribose of DNA-rN_1_-DNA/DNA is always missing in HIII, which may cause RNases HIII not to hydrolyze DNA-rN_1_-DNA/DNA substrates (Rychlik et al., 2010). It is surprising that a study on RNases HIII from *Chlamydophila pneumoniae* first discovered that RNase HIII can efficiently cleave DNA-rN_1_-DNA/DNA substrates in the presence of Mn^2+^ (Lu et al., 2012b). Later it was proved that RNases HIII from other organisms such as *B. subtilis, Staphylococcus aureus*, etc. also show Mn^2+^-dependent activity on such substrates (Randall et al., 2018). These findings demonstrate that the tyrosine residue conserved in RNases HII may not be the only way for RNases H to bind the single ribose in DNA-rN_1_-DNA/DNA substrates, as there is no such a tyrosine in RNases HIII. A serine residue in *C. pneumoniae* RNase HIII is involved in the recognition of the single ribose in dsDNA (Lu et al., 2012a; Hou et al., 2012b), but this residue does not seem to be conserved among HIIIs. The mechanism underlying the cleavage of RNase HIII on dsDNA containing a single ribose is therefore still unclear.

Additionally, both HII and HIII cleave Okazaki fragment–like substrates *in vitro*, but HIII may be more important in Okazaki fragment maturation *in vivo*, as shown in *B. subtilis* (Randall et al., 2019).

## Biodiversity of RNase H combinations

The genome of an organism usually contains more than one RNase H coding gene (*rnh*). For example, *B. subtilis* 168 and many pathogenic microbes contain three *rnh* genes, *E. coli* contains *rnh*A and *rnh*B, and archaea generally only have *rnh*B (Itaya et al., 1999; Kochiwa et al., 2007). Eukaryotes such as humans and yeast contain genes coding RNase H1 and RNase H2 (three subunits; Jeong et al., 2004; Cerritelli and Crouch, 2009; Chon et al., 2009; Shaban et al., 2010). Kochiwa et al. analyzed the number of *rnh* genes in 235 bacteria and 27 archaea, finding differences in combinations of *rnh* genes among individuals (Kochiwa et al., 2007). Is the diversity in *rnh* genes in individual organisms related to evolutionary relationships among living things? Tadokoro et al. did not think so (Tadokoro and Kanaya, 2009). *Shewanella* sp. sib1 and *Shewanella oneidensis* MR-1 are quite close in terms of evolution, but the former has three genes encoding RNases H and the latter has only two (Tadokoro et al., 2007). *T. maritima* and *Aquifex aeolius* are both superthermophilic bacteria and are related evolutionarily, but the former has the RNase H combination HI and HII whereas the latter has HII and HIII (Ohtani et al., 1999b). These findings suggest that during the process of evolution, different combinations of RNase H genes have become correlated with genetic relationships among organisms, and coding genes have been transferred horizontally among various branches.

The physiological significance of diverse combinations of RNase H genes is still unclear. However, the fact that RNases HII/H2 cut DNA-rN_1_-DNA/DNA substrates demonstrates that they participate in removing single ribonucleotides from genomic DNA. Type 1 RNases H cannot cleave such substrates, which indicates that HI/H1 does not play the same role in cells. Moreover, both type 1 and type 2 RNases H cleave RNA/DNA heterozygous substrates, so after a lethal mutation of one RNase H, the cell still has another RNase H to execute the same function to avoid cell death due to RNase H inactivation. This suggests why there are multiple RNase H genes in biological individuals.

Active RNases HI and HIII rarely coexist in the same genome (Kochiwa et al., 2007). An organism containing all three RNases H coding genes—*rnh*A, *rnh*B, and *rnh*C—such as *B. subtilis* 168, has no RNase H activity in HI (Chon et al., 2004). In terms of enzymatic characteristics, RNases HI and HIII have common features. For example, when they cut RNA/DNA or RNA–DNA/DNA substrates, their cleavage efficiency is usually greater than that of RNases HII, but except for a special subset of RNases HIII, neither can cleave DNA-rN_1_-DNA/DNA substrates (Tadokoro and Kanaya, 2009; Lu et al., 2012b; Randall et al., 2018, 2019). This suggests that the combination HI and HIII does not appear in a single genome because the functions of the two enzymes overlap, and the coexistence of HI and HIII may make RNase H function redundant. Organisms therefore chose one and abandon the other after evolving.

Although there is general agreement that RNases HI and HIII are partially redundant during biological evolution, a recent report found that the ancestral *B*. *subtilis* strain NCIB 3610 maintains all three functional RNase H proteins, with the combination of the chromosomally encoded HII and HIII plus an endogenous plasmid-encoded active HI (Nye et al., 2021). This implies that RNases HI and HIII can coexist in organisms, especially perhaps in those that appear in the early process of life evolution.

**Figure 3:**
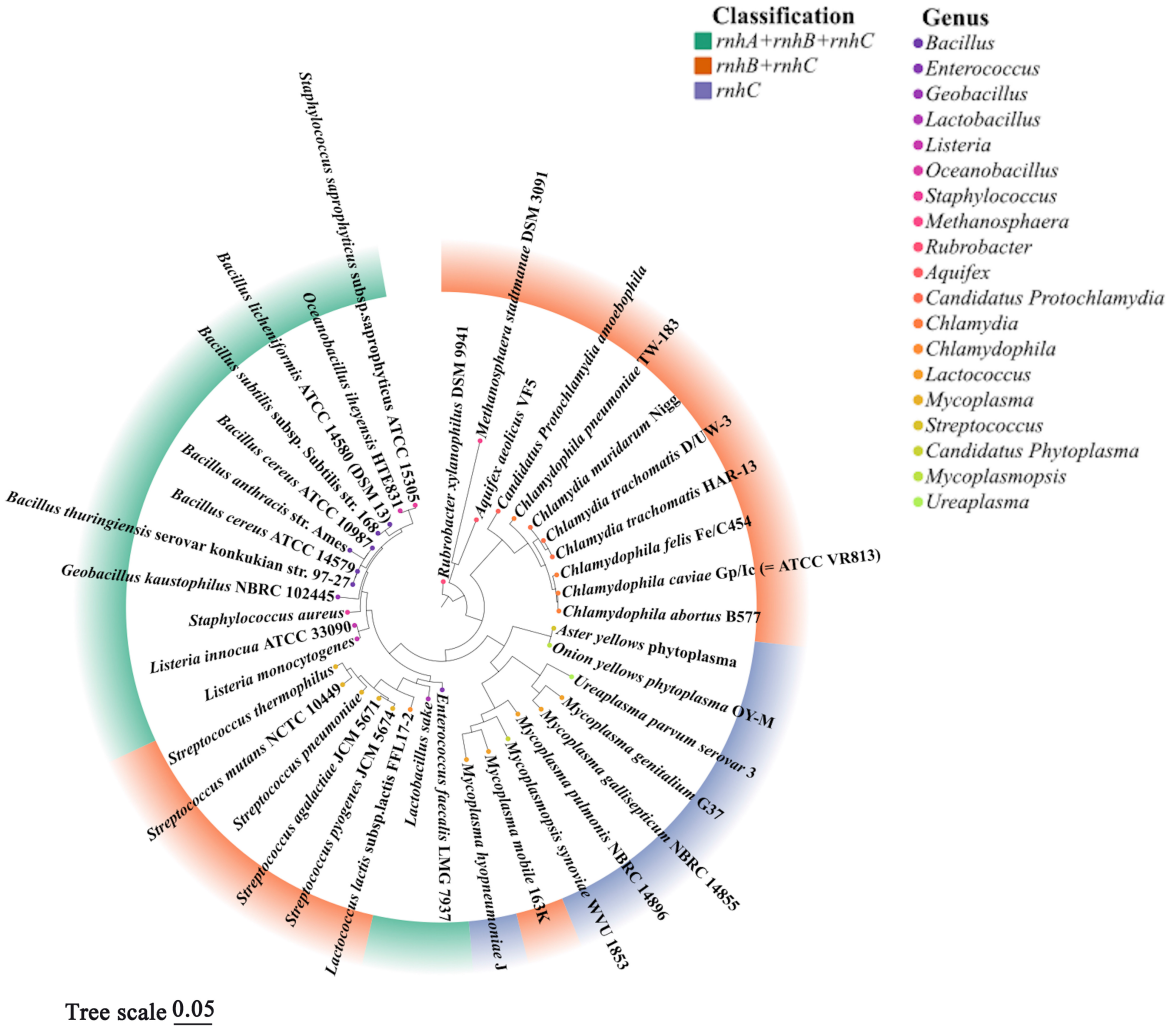
Phylogenetic tree of representative prokaryotes harboring *rnh*C gene. According to previous literature (Kochiwa et al., 2007) and the conservation of 16S rRNA, 40 prokaryotic organisms containing *rnh*C gene were chosen to align 16S rRNA sequences by CLUSTAL X2, a phylogenetic evolutionary tree was further built using MEGA 11.0 by the maximum likelihood, and the confidence limits were estimated by repetitive analyses of 1,000 bootstrapping replicates. Different colors in the outer circle of the diagram represent combinations of *rnh* gene, colors of points in the inner circle denote the genera of organisms. It is worth noting that not all *rnh* genes encode active RNase H, the fact that strains contain three *rnh* genes (ABC) does not mean these organisms always show activities of RNase HI, HII and HIII. This figure analyzed the evolutionary correlation of organisms according to the combinations of *rnh* genes but not the classification of active RNases H.

Two microorganisms representing the earliest branches of the biological evolutionary tree, *T. maritima* and *A. aeolius*, have varied combinations of RNases H, this seems to reflect the oldest state of formula for the combination of RNases H in the field: either HI and HII or HII and HIII. Interestingly, the coding genes RNases HI and HII exist widely in the biological world, but *rnh*C is only preserved in a few microbial populations; apparently it has been replaced by other RNases H genes in the long-term evolution process, RNase HIII is perceived to be redundant, and cells have avoided inheriting it as a part of evolution (Kochiwa et al., 2007). However, almost all *rnh*C genes exist in the prokaryotes which may undergo a host-inhabiting lifestyle at stages, such as strains of the genera *Staphylococcus*, *Chlamydophila*, *Mycoplasmas*, *Enterococci*, and so on (Figure 3; Kochiwa et al., 2007; Lu et al., 2012b). This implies that RNase HIII is probably necessary for these livings to colonize the host. *C. pneumoniae* RNase HIII can execute cleavage activity on DNA-rN_1_-DNA/DNA in an Mn^2+^-rich environment in which RNase HII activity is arrested. Thus, RNase HIII may function as a substitute for RNase HII in special physiological states, such as during fluxes of trace elements in infected organs, which is a normal response of an animal host to cellular pathogens (Lu et al., 2012b).

In Figure 3, based on the 16S rRNA gene sequences of representative prokaryotic organisms containing *rnh*C gene, we constructed a phylogenetic tree to analyze the evolutionary relationships among these livings. The result shows that organisms with the same combination of *rnh* genes are relatively convergent in the tree, suggesting they are close in evolution, such as *Staphylococcus aureus* and strains of *Bacillus* spp. The bacteria containing *rnh*A, *rnh*B and *rnh*C are more evolutionarily related to those harboring a single *rnh*C gene than those with a combination of *rnh*B and *rnh*C. This phylogenetic analysis emphasizes the correlation of biological choices of retaining different combinations of *rnh* genes with the genetic phylogeny.

In addition, a recent study suggests more functions of RNase HIII and the enzyme is not redundant for many prokaryotes. It shows that *rnh*C inactivation significantly increases cell viability of the Pcr A helicase-depleted *B. subtilis* cells, and it is further proposed that RNase HIII participates in R-loop-removement in *B. subtilis* (Moreno-Del Álamo et al., 2021).

Functional redundancy also exists in RNases HI/H1. At present, only a few type 1 RNases H with a heterozygous linkage domain (HBD) have been reported. However, analysis of an evolutionary model suggests that it is likely that type 1 RNase H with HBD and the enzyme without HBD were once present together in the same organisms and were partially redundant in the process of evolution (Kochiwa et al., 2007).

## Is RNase H essential for cell growth?

Researchers have long debated whether rnh is an essential gene for cell growth. As early as Kanaya and Crouch, (1984) proved that RNase H is necessary for growth in *E. coli*, as the bacteria will die after insertional inactivation of rnh, however, Itaya (Itaya et al., 1999) confirmed that when both RNase H genes are inactivated in *E. coli*, the bacteria grow normally, therefore, rnh genes are not essential in *E. coli*. In *B. subtilis*, each coding gene of RNases H can also be disrupted, although rnhBypeP double mutant forms small colonies, rnhBrnhC mutant shows rather smaller colonies and exhibits filamentous cell morphology (Fukushima et al., 2007). Based on these results, it was concluded that RNase H activity is dispensable for viability of *B. subtilis*, but the enzymes are responsible for the processing of RNA primers, rnh mutations can accumulate Okazaki fragments and induce SOS responses (Fukushima et al., 2007). More recently, a detailed survey of the role of RNase H in the survival of Mycobacterium smegmatis indicated a clear division of labor for mycobacterial RNase H enzymes, the growth rates of rnhA, rnhB or rnhC single mutants are similar with the wild type, but rnhA and rnhC were not able to be deleted simultaneously. The conclusion is that RNase HI activity, which can be provided by the enzyme coded by rnhA or rnhC, is essential for the growth of M. smegmatis, RNase HII (coded by rnhB) is dispensable (Richa Gupta et al., 2017).

In eukaryotes, the necessity of RNases H also varies from species to species. In one study, Rnaseh1-null mouse embryos are developmentally delayed and smaller at first but develop normally in gross morphology, then, they stop increasing in size and are mostly resorbed. This turning point coincides with the sharp decrease in the number of mitochondrial DNA, thus, RNase H1 is believed to be essential for mitochondrial DNA replication (Cerritelli et al., 2003). According to the result that a delay in development did not appear until the last larval instars, Filippov et al. agreed that RNase H1 is required for metamorphosis in Drosophila melanogaster but is not necessary for proliferation (Filippov et al., 1997, 2001). Mutations in human RNase H2 is a common cause of the genetic autoimmune disease—Aicardi-Goutières syndrome (AGS; Crow et al., 2006), there is accumulation of abnormal nucleic acids in the cell of AGS patients, and this causes constitutive inflammatory responses and in turn affects neurological function. The accumulated nucleic acids derive from recently replicated DNA, which indicates that the nucleases involved in AGS are related to DNA replication. Ray and Hines, (1995) found that deleting Rnaseh1 from Crithidia fasciculata did not affect mitochondrial functions. In another study, without RNases H, *Saccharomyces cerevisiae* did not die; the mutant lacking RNase H1 or RNase H2 was only highly sensitive to hydroxyurea, caffeine, and ethyl methane sulphonate (Arudchandran et al., 2000). This result confirms that RNases H play an important role in DNA replication and repair but that deletion of RNases H will not cause cell death in yeast. Moreover, Qiu et al. knocked out RNase H (35) in *S. cerevisiae*, and cell growth was not affected (Qiu et al., 1999b). Lazzaro et al. deleted RNase H1 and the three subunits of H2 in yeast one by one and found that each mutant could grow but showed different levels of replication pressure (Lazzaro et al., 2012).

In sum, RNases H have an important impact on cell growth, especially DNA replication and repair. Deletion of the enzymes causes disease or death in mammals but does not result in death in unicellular organisms such as bacteria and yeast.

## Unusual enzymes in the RNase H family

In the vast RNase H superfamily, a few RNases H appear to be unusual in their biological origins, enzymatic properties, or substrate hydrolyzation. If identified based on these characteristics, those enzymes are not typical RNases H; however, from the perspective of protein sequence or structure, they do belong to the RNase H family.

Almost all sequenced archaeal genomes contain only one RNase H: RNase HII. However, Ohtani et al. (Ohtani et al., 2004b) reported a special case in 2004: The halophilic archaeon *Halobacterium* sp. NRC-1 has a protein showing amino acid homology (33 and 34%) with *E. coli* and *B. subtilis* RNases HI, respectively. Biochemical analysis and an examination of functional complementation confirmed that the protein has RNase H activity (Ohtani et al., 2004a,b). This is the first example of archaea containing a type 1 RNase H, and it implies that other archaea not yet tested may have RNase HI too. Unlike normal RNases HI, *Halobacterium* sp. NRC-1 RNase HI (*Halo*-RNase HI) can nick at the 3′-end of the ribose of the RNA–DNA junction. That is, RNA primers in Okazaki fragments can be removed without leaving a ribonucleotide on the DNA strand. Compared to the typical type 1 RNases H, *Halo*-RNase HI lacks the catalytic-site histidine residue and the basic protrusion region involved in substrate binding. The mechanism underlying its catalysis is peculiar (Ohtani et al., 2004b).

Another archaeon, the thermophilic *Sulfolobus tokodaii*, has an RNase HI with unique activity: It can cleave dsRNA *in vitro* (Ohtani et al., 2004a). Currently, this is the only reported case of RNases H affecting dsRNA. A site-mutagenesis analysis showed that a mutation on Asp_125_ greatly affected the dsRNA cleavage activity of *Sto*-RNase HI, but the canonical RNase H activity was not significantly reduced, which suggests that the catalytic sites required for dsRNA cleavage differ from those required for conventional RNase H activity (Ohtani et al., 2004a).

There is another case of archaeal RNase H that specifically cleaves the phosphodiester bond at the RNA–DNA junction in double-stranded nucleic acids. The RNase HII from the thermophilic bacterium *Thermus thermophilus* HB8 shows 44% homology with that of *E. coli* and contains highly conserved active-site residues of type 2 RNases H. However, *in vivo* complementary experiments showed that the protein does not function as an RNase H. Moreover, *in vitro* experiments confirmed that it does not cleave RNA/DNA hybrids (the most common substrate of all RNases H) but only cleaves double-stranded nucleic acids with an RNA–DNA junction. As long as the substrate contains such a junction, regardless of whether it is dsDNA or dsRNA, it can be incised by this enzyme. This activity is different from canonical RNase H hydrolyzation, called junction ribonuclease (Ohtani et al., 2008).

## Conclusion

After decades of study, there are hundreds of reports revealing structures and functions of RNases H from various organisms. The mechanisms underlying RNase H catalysis and substrate recognition have become increasingly clear. At present, it is already difficult to find new in the mechanistic study solely through *in vitro* assays of RNases H, and so researchers have directed their attention to *in vivo* investigations, especially for RNases H derived from eukaryotic sources. As Cerritelli and Crouch noted (Cerritelli and Crouch, 2009), critical questions are what substrates RNases H act on *in vivo* and what roles different types of RNases H play in cells. A few researchers have attempted to investigate the substrate specificity of RNases H *in vivo*, but these questions are not yet completely answered.

Although Mg^2+^ and Mn^2+^ are divalent cations with similar chemical properties, many enzymes that catalyze phosphoryl transfer reactions prefer only one of them as a cofactor. There is no conspicuous difference in the crystal structures of the same protein complexed by Mg^2+^ and Mn^2+^, and the octahedral coordination geometry and ligand distance of both ions in the crystal structure are also the same (Rychlik et al., 2010). Although Yang and Nowotny have followed this question with interest since 2006 (Nowotny and Yang, 2006; Yang et al., 2006), a complete explanation is still lacking.

The mechanistic studies of RNases H provide a solid foundation for application. Inhibiting or blocking RNase H activity can prevent reverse transcription and render the HIV retrovirus unable to replicate. This is a reasonable strategy for treating this kind of viral disease. In addition, RNases H have been used in multiple antisense oligonucleotide technologies. RNase H1 is regarded as the gold standard for detecting R-loops and has been used to uncover the physiological and pathological roles of R-loops. More new applications will certainly develop that we cannot yet even dream of.

## Author contributions

JP, QG, and ZL collected data and wrote the article. Z made pictures. JP and QG prepared the table. All authors contributed to the article and approved the submitted version.

## Funding

This work was supported by the National Natural Science Foundation of China (no. 31970101), the Natural Science Foundation of Guangdong Province (no. 2019A1515011685) and the Research Start-up Funding of Shantou University (NTF18018).

## Conflict of interest

The authors declare that the research was conducted in the absence of any commercial or financial relationships that could be construed as a potential conflict of interest.

## Publisher’s note

All claims expressed in this article are solely those of the authors and do not necessarily represent those of their affiliated organizations, or those of the publisher, the editors and the reviewers. Any product that may be evaluated in this article, or claim that may be made by its manufacturer, is not guaranteed or endorsed by the publisher.
